# Attempting Measurement of Psychological Attributes

**DOI:** 10.3389/fpsyg.2013.00075

**Published:** 2013-02-26

**Authors:** Thomas Salzberger

**Affiliations:** ^1^Marketing, Vienna University of Economics and BusinessVienna, Austria

**Keywords:** measurement, construct validity, content validity, construct theory, Rasch model

## Abstract

Measures of psychological attributes abound in the social sciences as much as measures of physical properties do in the physical sciences. However, there are crucial differences between the scientific underpinning of measurement. While measurement in the physical sciences is supported by empirical evidence that demonstrates the quantitative nature of the property assessed, measurement in the social sciences is, in large part, made possible only by a vague, discretionary definition of measurement that places hardly any restrictions on empirical data. Traditional psychometric analyses fail to address the requirements of measurement as defined more rigorously in the physical sciences. The construct definitions do not allow for testable predictions; and content validity becomes a matter of highly subjective judgment. In order to improve measurement of psychological attributes, it is suggested to, first, readopt the definition of measurement in the physical sciences; second, to devise an elaborate theory of the construct to be measured that includes the hypothesis of a quantitative attribute; and third, to test the data for the structure implied by the hypothesis of quantity as well as predictions derived from the theory of the construct.

## Introduction

The quantitative imperative (Michell, 1999) dominates the social sciences, humanities, health, and related academic disciplines alike. Accordingly, measurement pervades the non-physical sciences as much as it does the physical sciences. But the success of measurement outside the natural sciences consists largely in hiding its Achilles heel: the lack of empirical evidence that the attributes measured are actually quantitative and, hence, measureable. Feynman (1981), when elaborating on pseudoscience, said “social science is an example of a science which is not a science; they don’t do [things] scientifically, they follow the forms (…)[but] they don’t get any laws, they haven’t found out anything.” Today, more than 30 years later, the social sciences are still in a struggle with countering Feynman’s harsh characterization of the social sciences. The following considerations largely characterize the situation in empirical marketing research, which strongly draws on psychological research.

Latent variables play a key role in the formulation and the testing of quantitative theories that suggest causal relationships. The proper measurement of latent variables is a pillar without which these theories would be untestable. However, the suggestion of a latent variable as a quantitative property is in itself a scientific theory. The theory claims the existence of an attribute that comes in degrees that can be measured (Michell, 1999; Borsboom et al., 2003; Borsboom, 2005). If the attribute exists as a quantity, it is, in principle, possible to state magnitudes of that attribute as a scalar times a unit of measurement, which is itself a particular magnitude of that attribute (Michell, 2004). In contrast to physical properties like length, mass, or temperature, psychological attributes are rarely, if at all, supposed to have a natural origin. Attitude toward a brand, for example, can vary between minus infinity and plus infinity. We are primarily interested in differences between consumers with regard to attitude, or in differences between attitudes toward various brands. For a particular consumer, a magnitude of attitude expresses the distance between that consumer’s attitude and an arbitrary reference point, for example another consumer’s attitude or the mean of a reference group. However, unless the magnitudes are expressed in the same unit, they are not comparable neither within the same consumer (when related to different brands, for example) nor between different consumers.

## Measurement and the Problem of Validity

A scientific theory needs to make predictions that can be tested empirically (Popper, 1996). This applies to theories relating different variables as much as it applies to the theory that a particular variable exists as a quantitative attribute. However, the suggestion of a latent variable is hardly ever framed as a theory, and no predictions are made that would indicate the quantitative character of the attribute. Consequently, no real empirical evidence is presented as to the theory of a quantitative attribute. The measures of the variables are used and interpreted as if they represented magnitudes that parallel those of measurement in physics. The problem of measurement is turned upside down inasmuch as the measurement of an attribute implies its existence as a quantity. Measurement of this sort is completely detached from the ontological claim of a quantitative latent variable. Rather, measurement “is defined as the assignment of numerals to objects or events according to rules” (Stevens, 1946, p. 677). Stevens does not specify the rules explicitly but refers to permissible transformations: “In what ways can we transform its values and still have it serve all the functions previously fulfilled?” (p. 680).

In practice, this reasoning results in a circularity as the scale level implies permissible transformations, which, in turn, determine the scale level. In case of metric scales, numerals are interpreted as numbers that “represent aspects of the empirical world” (p. 677). In the traditional paradigm of classical test theory (Lord and Novick, 1968), statistical analyses basically investigate the behavior of the presumed measures. Measurability is demonstrated by the fit to a factor analytic model and a reliability estimate beyond an agreed-on minimum level. The idea of formative indicators does not even require any sort of associations between item scores at all (Salzberger and Koller, 2012). Failure to demonstrate the suitability of a measurement instrument is almost exclusively attributed to an inadequate instrument. The very existence of the latent variable is hardly ever questioned. Others go even further and argue that “a ‘construct’ is a *definition* [and a] definition can be judged as reasonable or unreasonable but *not* as true or false” (Rossiter, 2011, p. 13, emphasis in the original). In other words, the category of trueness does not apply to latent variables, and, consequently, there is no need for empirical evidence of the actual existence of an attribute as a quantitative property. Whether a construct definition is reasonable or not is to be judged by experts based, at best, on criteria of logical consistency but not based on trueness. Rossiter (2011, p. 14) concludes that only a measurement instrument “can be validated–in relation to the construct as defined.”

If validity is reduced to agreement among experts framed as content validity, then one has inevitably left the realist camp. The meaningfulness of measures that are valid only with respect to an agreed-on construct definition is restricted to a socially constructed universe. The problem is that the term *valid* is linked to *reasonable* as well as to *true* and *real* (Merriam-Webster, 2012). From a realist point of view, the element of trueness is essential. However, subscribing to realism but not providing empirical evidence of trueness ultimately leads to the same consequences as Rossiter’s view on validity.

Validity based on agreement leads to paradoxical consequences when adhering to realism. There once was a time when the atom was considered the smallest and indivisible unit of matter. In fact, subatomic particles are a contradiction in terms given that atom literally means it cannot be cut. This also demonstrates that linguistic subtleties are not helpful. The terms reflect the state-of-affairs at a particular point in time but lose the literal meaning once the underlying theory is disproved. Today, we know that atoms are divisible and the fact that once everyone agreed on its indivisibility does not mean that the theory was ever right.

Rossiter (2011) views content validity as exhaustive. It is given by the semantic correspondence between a set of items and the conceptual definition of the construct. Any statistical modeling of data is at best unnecessary and in the worst case misleading, Rossiter (2011) argues. Indeed, one wonders why we actually have to demonstrate a particular behavior of scores which are at any rate measures by virtue of the assignment of numbers to response options and consequently to a respondent’s level of an attribute? The assignment of numbers is essentially an act of coding or scoring manifest responses. But following Stevens (1946, 1951), coding implies measurement. The scale level basically is a presumption researchers agree on.

If the existence of a quantitative attribute lies at the core of any attempt to measure a latent variable, traditional procedures of assessing validity miss the point. Convergent validity parallels internal consistency. It is concerned with the associations between scores of different instruments claiming to measure the same latent variable, while internal consistency addresses the relationships of individual items presumably related to the same concept. Other instruments, if available, typically lack themselves the evidence of measuring a quantitative attribute. The same problems persist when a proposed measure is related to antecedents and consequences in a nomological network (Cronbach and Meehl, 1955). Nomological validity relates a proposed measure to other measures whose validity is equally questionable. The scientific laws that give rise to the nomological network are conjectures that require validation in the first place. Borsboom et al. (2009) argue that in psychology no such laws have ever been established. Feynman’s (1981) view still holds true.

## Deficiencies of Current Practice

The disturbing state-of-affairs of measurement in the social sciences can be illustrated by the unrealistically high success rate, the lack of a measurement unit, the fragmentation of instruments that purport to capture the same latent variable, and the inadequate concept of content validity.

First of all, the success rate of asserted measurement approaches 100%. Even if one takes into account that the available evidence of measurement is affected by a publication bias, this record is remarkable. This is particularly astonishing given that we deal with the most complex system known to science – humans. Latent variables, like attitude or satisfaction, are arguably more complex than length or temperature. And still the measurement of psychological attributes does not seem to be more challenging than measuring physical attributes. In actuality, the overwhelming success rate of measurement in the social sciences is entirely due to Stevens’ non-committal definition.

Second, the measurement of psychological attributes consistently lacks a fundamental characteristic of measurement in the physical sciences: a common unit. The measurement of physical attributes has been unified, and a common metric has been established. Length, for example, is measured using the meter as the common (SI) unit. Measures expressed in other units can easily be converted. In the case of psychological attributes no common framework exists. The situation seems reminiscent on the primordial measurement of length where every sovereign defined a particular unit of measurement. At present, in the social sciences there are no real units at all. Pseudo-units are an incidental by-product of measurement by assignment. With factor scores, the unit is defined by the standard deviation of respondent scores. Apart from being sample-dependent and distribution-dependent, this unit is hard to interpret. As a consequence, researchers often resort to the average item score and rely on its interpretation with reference to the response categories. But this approach does not allow for a characterization of a particular level of the latent variable either. The challenge is therefore not just the unification of different units but the establishment of a proper unit in the first place.

Third, the absence of a common measuring system due to the lack of an established measurement unit also contributes to the fragmentation of measurement instruments and suggested attributes. Scales claiming to measure the same construct cannot be consolidated. As a consequence, generalizations at the level of structural theories relating different latent variables are severely impeded. On the other hand, scales that do capture the same latent variable may easily be taken to measure distinct latent variables. In the end, this indeterminacy is a consequence of measurement being essentially data-driven and bound to a particular frame of application rather than to a theory-driven concept definition.

Fourth, the theories of latent variables are rudimentary impeding validity assessment. Scale validation strongly emphasizes construct validity assessment based on convergent validity, discriminant validity, and nomological validity (Borsboom et al., 2004; Michell, 2009a). None of these validity aspects is related to the ontological claim entailed by the (mostly implicit) theory of the construct (see Borsboom et al., 2009). Content validity is equally blind to the fundamental question whether a latent variable exists as a quantity or not. Currently, suggested constructs are defined verbally on a very general level, but elaborate theories of constructs are extremely rare, if they exist at all. Content validity does not allow for theory-driven predictions under the assumption of a quantitative attribute that are empirically testable. Designing measurement instruments without evidence of the existence of the attribute as a quantitative latent variable is like taking the second step before ever having taken the first. In Michell’s (1999) terminology, we concern ourselves with the instrumental task of measurement, while we have not yet tackled the more fundamental scientific task of measurement (Michell, 1997).

To sum up, proper measurement requires three ingredients that are currently missing on the agenda of measurement: first, the definition of measurement in the physical sciences has to be readopted; second, an elaborate theory of the construct has to be developed that includes the hypothesis of a quantitative attribute; and third, the empirical analysis has to test the data for the structure implied by the hypothesis of quantity and it has to test the predictions derived from the theory of the construct.

## Milestones Toward Measurement

The fundamental prerequisite of proper measurement of psychological attributes is to adopt the definition of measurement maintained in the physical sciences so that the definition is unified across the sciences. It appears to be the easiest step. But since it has far-reaching consequences that cannot be dismissed as “only philosophical”, it requires commitment and one has to expect fierce resistance. While it is merely the acknowledgment of the scientific goal of quantitative research, it overthrows more than half a century of quantitative empirical research (see Michell, 2000). The development of a more comprehensive theory of a quantitative construct lies at the core of measurement but is a challenge that can hardly be underestimated. Finally, a measurement model has to be chosen that accounts for the requirements of measurement. The choice is primarily determined by the consequences of the definition of measurement. While Stevens’ definition of measurement hardly places any restrictions on the properties of the measurement model, the unified definition implies well-defined constraints.

Measurement understood as the quantification of a latent variable that actually exists as a quantitative property implies a structure in the data that ultimately follows from the axioms of quantity (Michell, 2009b) and, specifically, the theory of simultaneous, or additive, conjoint measurement (Luce and Tukey, 1964). Consequently, a measurement model has to be sensitive to violations of that structure. Particularly, cancelation conditions have to be met (see Michell, 1990). The expected responses based on the Rasch model for measurement (Rasch, 1960) have been shown to comply with these requirements (Karabatsos, 2001). Kyngdon (2008), though, points out that this does not necessarily imply that the Rasch model is a practical realization of additive conjoint measurement as a way to demonstrate additivity. From a more pragmatic point of view, empirical fit of data to the Rasch model at least lends credence to the successful measurement of a quantitative latent variable. Moreover, the Rasch model requires invariance (Andrich, 2004) as a consequence of specific objectivity (Rasch, 1961, 1977). If invariance is empirically supported, a wide frame of reference can be established facilitating generalization.

In contrast to traditional test theory and item response theory (IRT; Embretson and Reise, 2000), which fails to comply with cancelation conditions (see Karabatsos, 2001) and does not entail invariance, the Rasch model is linked to the theoretical requirements of quantity and, thus, of measurement. Fit of data to the Rasch model is therefore much more informative than fit to factor analytic models or IRT. However, the Rasch model is an intrinsically confirmatory model. It requires a solid theoretical foundation in terms of the suggested latent variable. If such a theoretical underpinning is missing, the application of the Rasch model becomes an essentially exploratory undertaking. This is particularly true if one is willing to discard a large number of items that fail to meet the criteria of fit. Fit of the data to the Rasch model is therefore a necessary but not a sufficient condition. The interpretation of measures has to be rooted in theory. Therefore, a proper substantive theory of the construct is a *sine qua non*. The theory should allow for an expected structure in the data. At least the order of manifest items should be theory-based allowing for a comparison of expected and observed patterns. The ultimate goal of measurement has to be the investigation of the causal mechanism that drives item and person measures (see Stenner et al., 2009). Such a mechanism spells out what determines the location of a particular item and allows for concrete experimental manipulations. It remains to be seen whether this will ever be achievable in psychological measurement. But in any case the difficulties involved must not prevent us from trying.

It is hardly possible to underestimate the implications of a paradigmatic shift from one definition of measurement to another, from a plethora of descriptive measurement models to a much more rigorous prescriptive model like the Rasch model. However, the challenges in terms of construct theory building are probably even harder to master. As long as substantive theories of latent variables are not advanced enough, evidence of measurement based on the fit of data to the Rasch model should be interpreted with caution. On the other hand, science proceeds in a sequence of theory building and theory testing. The application of the Rasch model provides insight that can inform the theory of the construct under scrutiny. If the property of invariance is empirically confirmed by a series of successful replications of item measures across diverse conditions, this can be taken as an indication of a quantitative latent variable underlying the manifest responses.

Despite the obvious limitations of current practices of measurement in the social sciences, there is, at present, hardly any willingness to acknowledge the problem, which could not be more fundamental to quantitative research. Concerns are pushed aside and regarded as purely philosophical. Indeed, there is much at stake. Academic disciplines in the social sciences would need to rethink a good part of their body of empirical research. There seems to be more to lose than there is to win. But overcoming deceptive quantification of possibly non-existing latent variables should be viewed as an important scientific achievement in itself, not as a loss. The prospect of properly measured latent variables and established standards that allow for comparisons of measures derived from different instruments should be encouraging. In the mid-twentieth century, the adoption of Stevens’ definition of measurement safeguarded psychology against being rejected as a (quantitative) science (see Michell, 1999). Today, it is clear that the social sciences had to pay a high price: measurement has become an exercise that is completely detached from the very meaning of scientific quantification. Specifically, the understanding of measurement by assignment contradicts the self-concept of the social sciences as empirical sciences rooted in realism. Today, the rejection of the idea of measurement by assignment and the reversion to the unified definition of measurement in science are required to maintain the claim of being a quantitative science.

## Conflict of Interest Statement

The author declares that the research was conducted in the absence of any commercial or financial relationships that could be construed as a potential conflict of interest.
